# Genetic Variation in Transcription Factor Binding Sites

**DOI:** 10.3390/ijms24055038

**Published:** 2023-03-06

**Authors:** Gabriel Santpere

**Affiliations:** Neurogenomics Group, Research Programme on Biomedical Informatics (GRIB), Hospital del Mar Medical Research Institute (IMIM), MELIS, Universitat Pompeu Fabra, 08003 Barcelona, Spain; gabrielsantperebaro@gmail.com

The interaction between transcription factors (TFs) and DNA is the core process that determines the state of a cell’s transcriptome. Changes in TF binding can have consequences for normal cellular development as well as function and can also be the substrate for molecular evolution. Comparing TF–DNA binding across cells, tissues, and organisms, as well as across individuals, conditions, states, and developmental trajectories, is fundamental to assessing the impact of the gene regulatory networks’ (GRNs) dynamics on normal cellular function, disease, and evolution. There are many observed mechanisms by which a given TF–DNA binding event can be altered. For example, at the TF level, variations in the concentration, subcellular localization, and post-translational modifications of TFs and their cofactors can all help to explain such differences. At the DNA level, changes in chromatin status (e.g., histone modifications and DNA methylation) and sequence variations that alter the preferred motifs of TFs or DNA shape may be determinants of GRN variation. Indeed, a large body of evidence links genetic mutations that specifically disrupt binding sites not only to the expected differences in TF binding and gene expression [1] but also to the allelic imbalance in chromatin accessibility. This latter observation applies to both segregating sites within populations [2,3] and substitutions between species [4], and further illustrates the bidirectional relationships between TF binding and chromatin accessibility that play a role in, as well as determine, target gene expression differences. Not surprisingly, TFBS-disrupting variants are particularly enriched at GWAS loci [5] and represent the best candidates with which to explain the functional consequences of evolutionarily relevant genomic regions [6]. In this Special Issue, entitled “The Role of Genetic Variation in Transcription Factor Binding Sites in Evolution and Disease”, we aim to present and promote research on these aspects, which cut across a wide range of research areas. 

Degtyareva et al. review the history of regulatory SNPs that modify TFBS and discuss the range of available methods that can be used to dissect the specific TFs affected by genetic variants, such as EMSA, ChIP-seq, and pull-down assays [7]. To extend this type of analysis genome-wide, with the aim of prioritizing causal GWAS variants, the authors discuss the capabilities of current state-of-the art functional genomics methods, such as eQTL mapping, massive parallel reporter assays, and ChIP-seq, as they apply to the determination of allele-specific expression, regulation, and TF binding. Finally, the review provides illustrative examples of disease-associated variants from GWAS where TFs with allele-specific binding have been identified [7]. Another practical example of using allelic asymmetries in gene expression and chromatin state to identify regulatory SNPs is provided by Korbolina et al. In their study, the authors leverage RNA-seq and H3K4me3 ChIP-seq data from human pulmonary arterial endothelial cells to identify such allelic asymmetries, a catalogue that is subsequently refined with existing eQTL and GWAS data [8]. Tseng et al. complement this by reviewing aspects of the mechanisms that influence TF binding, including histone modifications, DNA methylation, and chromatin conformation, as well as providing several instances of disease risk alleles that affect the interplay between specific TFs and chromatin status [9].

The experimental determination of TF binding sites is an essential step as it reveals a variety of nuances in motif preference that often misalign with in silico predictions. In de Martin et al., the authors focus on the complexities of TF binding to DNA of the basic helix–loop–helix (bHLH) TFs, and how motif preference is determined by a combination of factors, including spatiotemporally regulated co-factor interactions, post-translational modifications, and chromatin status [10]. Members of the bHLH family are also the subject of Yoshikawa et al., who review the evolutionary history of the regulatory elements associated with the recombination-activating genes Rag1 and Rag2, which mediate the recombination process that confers variability to T cell receptors and immunoglobulin genes, as well as their bHLH regulators [11]. 

TFs can also operate on regulatory regions encoded in the genomes of DNA viruses. For example, hosts’ TFs initiate and coordinate the viral cycle of the JC polyomavirus (JCPyV), the causative agent of a demyelinating disease called progressive multifocal leukoencephalopathy (PML). Wilczek et al. present their work on the structural variation affecting the JCPyV regulatory region and how this affects host TF binding. Their study found that JCPyV rearrangements resulting in more TFBS of TFs enhancing viral replication are more common in PML than in non-PML samples [12]. Finally, Wang et al. introduce the topic of gene clusters regulated by few TFs and the grammar of their components. In particular, the authors review the complexity and interactions of pathway-specific TFs involved in the regulation of biosynthetic gene clusters in two model fungal organisms [13]. 

Taken together, this Special Issue illustrates a wide range of molecular mechanisms that generate variation in TF function in different organisms. It also reflects the importance of combining computational modeling and prediction with the experimental determination of TFBS. I hope that this Special Issue will contribute to drawing more attention and research to variation in TF–DNA interactions in the context of evolution and disease.
